# Semen May Harbor HIV Despite Effective HAART: Another Piece in the Puzzle

**DOI:** 10.1371/journal.pone.0010569

**Published:** 2010-05-13

**Authors:** Philippe Halfon, Claude Giorgetti, Hacène Khiri, Guillaume Pénaranda, Philippe Terriou, Géraldine Porcu-Buisson, Véronique Chabert-Orsini

**Affiliations:** 1 Department of Virology, Laboratoire Alphabio, Marseille, France; 2 Institut Médical de Reproduction, Marseille, France; 3 Department of Molecular Biology, Laboratoire Alphabio, Marseille, France; 4 Department of Biostatistics, Laboratoire Alphabio, Marseille, France; Duke University Medical Center, United States of America

## Abstract

**Background:**

The risk of male-to-female intravaginal HIV-1 transmission is estimated at about 1 event per 200–2000 coital acts. The aim of this study was to assess the residual risk of HIV presence in semen in patients under HAART therapy.

**Methods and Findings:**

The study took place in France from October 2001 to March 2009. 394 paired blood and semen samples were provided from 332 HIV-1 infected men. The Roche Cobas AMPLICOR Monitor HIV assay was used to quantify HIV-1 RNA in blood and in seminal plasma. Three percent of 394 HIV-1 infected men enrolled in an assisted reproductive technology program harbored detectable HIV-1 RNA in semen, although they had no other sexually transmitted disease and their blood viral load was undetectable for at least 6 months under antiretroviral treatment.

**Conclusion:**

These data suggest that undetectable plasma HIV RNA means a lower risk of viral transmission through seminal fluid on a population level, but not necessarily at the level of the individual.

## Introduction

HIV-1, the causative agent of AIDS, has infected about 33 million people and caused over 20 million deaths (UNAIDS data). More than 80% of these HIV-1 infections are acquired through sexual intercourse. Despite its dramatic spread in the human population, the efficiency of HIV-1 transmission via the sexual route is surprisingly poor. For instance, the risk of male-to-female intravaginal HIV-1 transmission is estimated at about 1 event per 200–2000 coital acts [1]. Globally, most infections result from genital exposure to semen (SE) of HIV-positive men [2]. Women who acquired HIV-1 through vaginal intercourse constitute almost 60% of new infections in Africa [3]. Protected intercourse is strongly recommended for HIV-serodiscordant couples in all circumstances.

Some leading researchers have suggested that effective HIV treatment essentially renders a patient non-infectious. Previous data published by the Swiss Federal Commission for HIV/AIDS suggested that seropositive individuals, with no other sexually transmitted disease, on antiretroviral therapy, with undetectable viral loads for >6 months, do not transmit HIV [4]. In order to avoid the risk of HIV sexual transmission to women, when men are infected, HIV-serodiscordant couples now have access to assisted reproductive technology (AR) programs in several countries. These programs vary in the assisted reproductive technology methods chosen (intrauterine insemination, in-vitro fertilization, intracytoplasmic sperm injection) and in the type of sperm preparation used (density gradient migration alone or followed by swim-up). Good rates of pregnancy are reported when the male partner is infected (63%) and no seroconversion has been reported to date [5]. The infectiousness of HIV-1 in male genital fluid together with the susceptibility of the host, the type of sexual practice, and viral load are major determinants of sexual transmission [6]. The factors modulating HIV infectiousness in semen are poorly understood [7]. Several factors can interfere and may have the potential to increase risk, such as fluctuation of adherence, drug characteristics influencing penetration into compartments, and asymptomatic and undiagnosed STDs. Moreover, compartmentalization of HIV replication in semen has been demonstrated for some men and, therefore, HIV blood viral load might not always reflect HIV replication levels in semen [8], [9]. Although HAART reduces HIV loads in both blood and seminal compartments, low levels of HIV RNA can still be detected in seminal plasma and HIV-infected cells can be recovered in nonsperm cells, even in those who have undergone prolonged successful treatment [10], [11]. A recent study, demonstrated that HIV may still be present and potentially infectious in semen, even if it is undetectable in blood [12]. The aim of this study is to assess the residual risk of HIV presence in semen in patients under HAART therapy.

## Materials and Methods

### Patients

Since 2001, our centre has managed HIV-1-serodiscordant couples with a male infected partner to allow pregnancies with assisted reproduction using sperm washing. Three hundred and thirty-two HIV-1 infected men attending the Laboratory IVF (Marseille, FRANCE) were included in the analysis with respect of the French law (no HIV related active disease, a regular follow up and CD4 counts >200 mm3 repeated at least twice during the last 6 month and a blood viral load <10 000 copies/ml stable during the last 6 month) after they gave their fully informed consent. Characteristics of patients were: mean age ± Sd 39±4 years, HIV mode of transmission (54% intravenous drug use, 41% sexual, and 5% blood transfusion), median known treatment duration, 11.5 years.

As this study included only patients issued from routine follow up, declaration to ethic committee is not mandatory, as recommended by the French Government Rules.

### Samples

Overall 394 paired blood and semen samples were provided between October 2001 and March 2009 (median time difference between blood and semen samples was 28 days). The number of samples provided by the patients varied between one to four. Plasma samples were separated from blood by centrifugation and frozen at −80°C until use. After 2–5 days of sexual abstinence, semen samples were obtained by masturbation into a sterile container and were processed within 1 h of ejaculation. If available one milliliter of the semen sample was centrifuged at 800× g for 10 min through a two-layer discontinuous gradient (2 ml of 40, and 90% of SupraSperm, Medicult, France). The seminal plasma (supernatant) was separated and stored at −80°C until further use. The motile spermatozoa fraction were recovered from the 90% fraction and washed once with an equal volume of BM1 medium (Eurobio, France). The resulting centrifugation pellet was resuspended and observed by microscope to count them and to check for the absence of white blood cell contamination. This motile spermatozoa fraction was frozen at −80°C as aliquots of 500,000 cells until use.

### Detection of HIV RNA in blood plasma

HIV RNA in blood plasma was detected by Cobas Taqman AMPLICOR HIV assay (Roche Diagnostics, Meylan, France), according to the instructions of the manufacturer. Sensitivity of the quantitative assay was 40 copies/ml (1.6 log)[13].

### Detection of HIV RNA in seminal plasma

RNA extraction was performed using silica beads (NucliSens [Organon Teknika S.A., Fresnes, France]) For HIV RNA we used 500 µl of thawed fraction 1 and 6.7 µl of the internal control from the Roche Cobas AMPLICOR Monitor HIV assay. The eluted sample was diluted with specimen diluent of Roche Amplicor Monitor HIV kit to obtain 100 µl of sample in a new tube.

The sensitivities of the quantitative assays were determined by using serial 2-fold dilutions (from 320 to 20 copies/ml) or 10-fold dilutions (from 4 000 to 4 copies/ml), respectively, in HIV-negative seminal plasma of blood plasma from an HIV-infected patient, which had been quantified previously by the Cobas AMPLICOR Monitor HIV assay. Then, each dilution was extracted and tested in six independent experiments. Sensitivity of the quantitative assay was 40 copies/ml (1.6 log) [13].

## Results and Discussion

**Figure 1:**
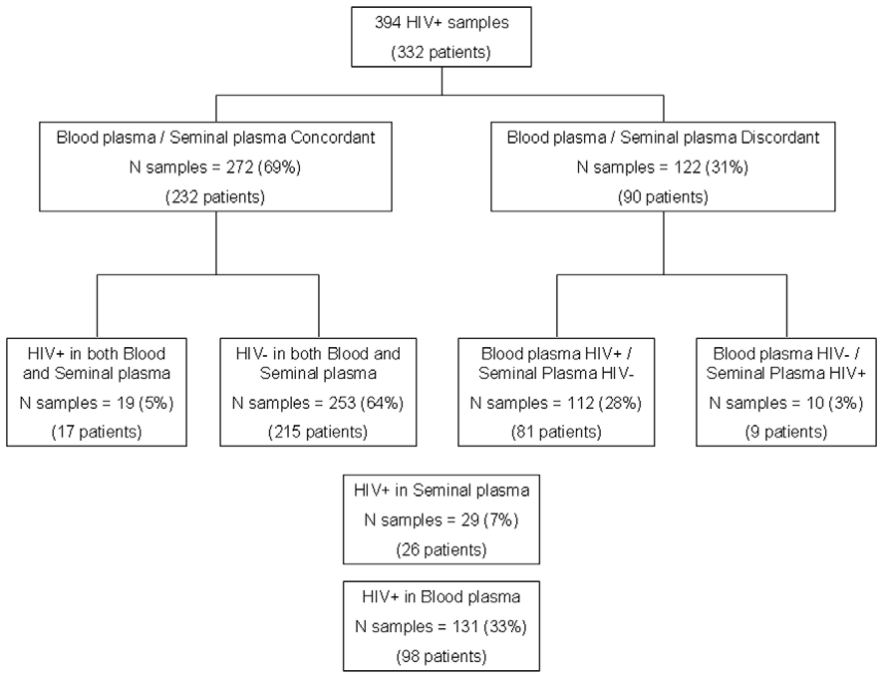
Study flowchart.

**Figure 1** represents the study flowchart. Among the 394 paired samples, HIV-1 was detectable in 131 (33%) blood plasma samples and the median level of HIV-1 RNA in blood was 468 copies/ml (range = 50–10 400). HIV-1 was detectable in 29 (7%) of seminal plasma samples and the median level of HIV-1 RNA in semen was 1360 copies/ml (range [73–435 000]). Overall, 272 (69%) paired samples were concordant between blood plasma and seminal plasma for HIV-1 detection (inter-rater agreement k = 0.12 [−0.01;0.25]); 253 (64%) samples were HIV-1 negative both in blood plasma and seminal plasma, and 19 (5%) samples were HIV-1 positive both in blood plasma and seminal plasma. Overall, 122 (31%) paired samples were discordant between blood plasma and seminal plasma for HIV-1 detection. Among these, 10 (3%) seminal plasma samples had detectable HIV-1 RNA although blood viral load was undetectable for at least 6 months under antiretroviral treatment (**Table 1**). Note that the median HIV-1 RNA seminal plasma viral load found in these 10 patients was low (median 1545, range [576–23 000]). This does not seem to be related to a specific treatment, because these patients were treated by various therapies comprising a non-nucleoside reverse transcriptase inhibitor or protease inhibitor-based regimen, with, in some cases, antiretroviral drugs known to have good penetration into genital compartments [14]. Note that diffusion of antiretroviral drugs into genital compartment is highly variable. NRTIs have a good penetration with plasma/genital secretion ratio around 100% and more for the most common used (3tc, ftc, tdf, abc, azt). Nevirapine present also a good penetration (around 80%) in comparison with efavirenz (<1%). Among PI, only IDV who presents a lower protein bound fraction had a higher plasma/genital secretion ratio (100–200%) than the others for which the ratio is below 50% and less. New drugs, such raltegravir and maraviroc appear to have a good penetration ratio (70 to more than 200%) in these compartments [14], [15].

**Table 1:** Characteristics of the 10 patients negative for HIV-1 blood plasma result and positive for HIV-1 seminal plasma.

Patient	Age (years)	CD4 cells (cells/µl)	HIV-1 RNA (cps/ml)		N° of circular cells (10^6^/ml)	Antiretroviral treatment
			Blood Plasma	Seminal Plasma		
1	43	940	<40	1420	6.0	ZDV **3TC**
2	40	498	<40	11 200	25.0	LPV RTV **3TC** SQV
3	34	521	<40	1230	0.0	ATV ZDV **3TC**
4	47	473	<40	963	7.0	ZDV **3TC** LPV RTV
5	43	314	<40	1670	1.0	LPV RTV **TDF** **FTC**
6	37	800	<40	3210	3.0	DDI D4T NFV
7	38	649	<40	23 000	6.0	D4T EFV NFV
8	33	386	<40	576	39.0	LPV RTV **TDF**
9	33	211	<40	18 670	1.0	EFV DDI LPV RTV
10	44	430	<40	770	7.0	ZDV **3TC**

3TC, lamivudine; ATV, atazanavir; D4T, stavudine; DDI, didanosine; EFV, efivarenz; FTC, emtricitabine; LPV, lopinavir; RTV, ritonavir; SQV, saquinavir; TDF, tenofovir; ZDV, zidovudine.

In bold are the antiretroviral with good penetration into genital compartment.

A recent study tried to link between the seminal viral load and HIV transmission. It indicated that the risk of HIV transmission from male to female is related to genital HIV-1 RNA concentrations. It was shown that the rate of HIV sero-incidence increased as well as the HIV seminal plasma concentrations increased: each increasing of HIV viral load of 1 log c/ml in the plasma seminal increased by ×1.85 the risk of HIV transmission [16].

Previous studies have already shown that intermittent HIV RNA shedding can occur in semen from treated patients with undetectable HIV viral load in blood [9], [13], [17]. Our results confirm those of Marcelin et al. on 257-paired blood and semen samples followed for greater than 6 months [12]. In this study, 225 were concordant with undetectable HIV levels in blood and semen and 9 had detectable levels of HIV in both blood and semen. Interestingly, 23 samples had HIV viral loads detectable in blood but not semen and 7 samples had undetectable viral load levels in blood but detectable virus in the semen (5%) (sensitivities of the assay for detecting HIV in blood and in semen were 40 cp/ml and 200 cp/ml respectively) [12]. Our results also confirm a study by Sheth et al. on 25 patients with undetectable blood HIV RNA measurements 16 weeks after HAART initiation (sensitivity of the assay for detecting HIV in blood was 50 cp/ml) [18]. Isolated HIV shedding in semen was detectable in 12 of 25 patients despite effective HAART therapy, and at a high level (>5000 copies/mL) in 4 of 25 participants. Semen isolates did not contain drug resistant virus and the HIV detected was infectious in vitro. The only measurement that seemed to predict the presence of persistent HIV in semen despite HAART was pre-HAART semen HIV levels. A recent finding is that Amyloidogenic PAP fragments are abundant in seminal fluid and boost semen-mediated enhancement of HIV infection. Thus, they may play an important role in sexual transmission of HIV [19]. Another recent study highlights the residual risk of HIV-1 transmission during unprotected intercourse and raises the question of the possible consequences of ineffective HAART in the genital tract [20].

In conclusion, between 3% (the present study) to 5% of patients with undetectable HIV levels in blood had detectable levels of HIV in semen [12]. These data, suggest that undetectable plasma HIV RNA means a lower risk of viral transmission through seminal fluid on a population level but not necessarily at the level of the individual.
